# Therapeutic targets and signaling mechanisms of dasatinib activity against radiation skin ulcer

**DOI:** 10.3389/fpubh.2022.1031038

**Published:** 2022-11-30

**Authors:** Wenxing Su, Xuelian Chen, Wen Zhang, Dazhuang Li, Xiaoming Chen, Daojiang Yu

**Affiliations:** ^1^Department of Plastic and Burn Surgery, The Second Affiliated Hospital of Chengdu Medical College (China National Nuclear Corporation 416 Hospital), Chengdu, China; ^2^Department of Cosmetic Plastic and Burn Surgery, The First Affiliated Hospital of Chengdu Medical College, Chengdu, China; ^3^School of Clinical Medicine, Chengdu Medical College, Chengdu, China; ^4^Department of Orthopedics, The Affiliated Hospital of Yangzhou University, Yangzhou University, Yangzhou, China

**Keywords:** dasatinib, radiation ulcer, molecular docking, network pharmacology, core target

## Abstract

**Objective:**

To reveal the potential targets and signaling pathways of dasatinib in the treatment of radiation ulcers through network pharmacology and molecular docking technology.

**Methods:**

Pathological targets of radiation ulcers were screened using GeneCards database. At the same time, the pharmacological targets of dasatinib were obtained through SwissTargetPrediction (STP), Binding DB and Drugbank databases. Subsequently, the potential targets of dasatinib for anti-radiation ulcers were obtained after intersection by Venn diagram. Next, a protein-protein interaction (PPI) network was constructed through the STRING database and core targets were screened. Finally, the identified core targets were subjected to GO and KEGG enrichment analysis, co-expression network analysis, and molecular docking technology to verify the reliability of the core targets.

**Results:**

A total of 76 potential targets for anti-radiation ulcer with dasatinib were obtained, and 6 core targets were screened, including EGFR, ERBB2, FYN, JAK2, KIT, and SRC. These genes were mainly enriched in Adherens junction, EGFR tyrosine kinase inhibitor resistance, Focal adhesion, Bladder cancer and PI3K-Akt signaling pathway. Molecular docking results showed that dasatinib binds well to the core target.

**Conclusion:**

Dasatinib may play a role in the treatment of radiation ulcers by regulating EGFR, ERBB2, FYN, JAK2, KIT, and SRC. These core targets may provide new insights for follow-up studies of radiation ulcers.

## Introduction

Radiation ulcers are the result of skin damage in excess of the cumulative radiation dose during radiation therapy or accidental exposure to radiation sources (1, 2), and the histological features of the lesions include epidermal atrophy, dermal sclerosis, superficial vasodilation, adnexal structures loss, etc. (3, 4). However, so far, there is no clear clinical consensus on the treatment of radiation ulcers. Conservative treatment has a significant effect on radiation dermatitis (without ulcers), but severe cases, especially ulcers, require surgical intervention to promote wound healing (5, 6). Therefore, it is particularly important to minimize radiation dose to prevent radiation ulcers (7–9). In addition, patients should also receive appropriate skin care education and regular dermatological monitoring and evaluation when at therapeutic radiation dose levels (7, 8). Animal experiments showed that no skin changes were seen in the 10 Gy irradiation group; mild skin damage was seen in the 15 Gy irradiation group; skin ulcers appeared in some rats in the irradiation group exceeding 20 Gy; the rats irradiated with 35 to 55 Gy could successfully Induced radiation-induced skin ulcer model (10–12).

Tissue reconstruction of radiation skin ulcers presents a great challenge for plastic surgeons. Anti-inflammatory drugs, growth factors, and local anesthetics are commonly used clinically to relieve radiation skin ulcers, but the effect is not good (13). Hyperbaric oxygen therapy is also considered an effective way to reduce skin ulcers after radiation therapy, but the treatment time is very long (14, 15). The pathological mechanism of radiation skin ulcers is unclear, and there is no effective treatment (16). Therefore, there is an urgent need for a safe and effective drug that can relieve radiation skin ulcers. Previous studies have confirmed that Cordycepin can prevent and treat radiation ulcers by inhibiting NRF2 and AMPK-induced cellular senescence (17). Therefore, senescent cells may be a viable target for the treatment of radiation ulcers (18). Dasatinib is a tyrosine kinase inhibitor first approved for the treatment of chronic myeloid leukemia (19). Kinases inhibited by dasatinib mainly include Src family kinases (SFK), cKIT, platelet-derived growth factor receptor, TEC and SYK families (19, 20). Recent animal studies have shown that dasatinib and quercetin can promote the healing of radiation ulcers by removing senescent cells (21). Dasatinib has shown potential to treat radiation ulcers, but its specific molecular mechanism remains unclear. Therefore, this study explored the potential targets and signaling pathways of dasatinib in the treatment of radiation skin ulcers through network pharmacology and molecular docking. Our findings provide a rationale for the mechanism of action of dasatinib in the treatment of radiation skin ulcers, which can be validated before being used in clinical trials.

## Methods

### Prediction of dasatinib pharmacological targets and pathological targets in radiation skin ulcer

The SMILES (Simplified Molecular Linear Input Specification) is obtained from the PubChem (https://pubchem.ncbi.nlm.nih.gov/) database with the search term “Dasatinib,” which is an ASCII string that explicitly describes the three-dimensional chemistry of molecules structure. Import the retrieved SMILES into the SwissTargetPrediction (http://www.swisstargetprediction.ch/) database and the Binding DB (http://www.bindingdb.org/bind/index.jsp) database, with the respective filter conditions set as Probability > 0 and Similarity > 0.85. Subsequently, in the Drugbank (https://go.drugbank.com/) database, its potential targets were searched with the keyword “Dasatinib,” and all its drug target sources were supported by corresponding experimental verifications. The above search species are all set to “homo sapiens.” Finally, the final dasatinib pharmacological targets were obtained after screening and deduplication of the targets obtained from the three databases. The pathological targets were obtained by searching with “radiation skin ulcer” as the keyword, and setting the target with score > 1 form GeneCards database (https://www.genecards.org/). This is a database that provides detailed information on all currently annotated and predictable genes in humans.

### Acquisition and analysis of dasatinib activity against radiation skin ulcer intersection target

The intersection targets of Dasatinib against radiation skin ulcer were obtained through the venn diagram (https://bioinfogp.cnb.csic.es/tools/venny/index.html), and then the intersection targets were imported into the STRING (https://cn.string-db.org/) (Version 11.5) data analysis platform for PPI analysis (species set to “homo sapiens,” protein interaction score set with a high confidence level of 0.400). Subsequently, the PPI network was visualized by Cytoscape (version 3.9.0) software. KEGG and GO analyses were performed on the intersection targets by using the online analysis tool Kobas 3.0. Subsequently, its results were visualized by R software.

### Identification and analysis of core targets

The six commonly used topological analysis methods (Closeness, Radiality, Degree, Betweenness, Stress, EPC) in Cytohubba, a plug-in of Cytoscape software, were used to identify the core targets of Dasatinib activity against radiation skin ulcer. Subsequently, the co-expression network and functional analysis of core targets were constructed through the GeneMANIA (http://www.genemania.org/) database, which is mainly used to generate hypotheses about gene functions and analyze gene lists. KEGG and GO analyses were performed on the core targets by Kobas 3.0. Subsequently, its results were visualized by R software. Finally, the Drug-target-disease network was drawn by Cytoscape software.

### Molecular docking verification-AutoDock vina

We enter the gene symbol of the core target in the PDB (https://www.rcsb.org/) database to obtain the protein structure crystal. In order to accurately obtain the PDB ID of the core target, the screening conditions are set as: Experimental method is set to X- ray diffraction, Refinement resolution is set to ≤ 2A, PH is selected to be 7.35–7.50, and chemical components are selected to have Ligand. We downloaded the 3D crystal structure of the core target from PDB database, selected to be saved in PDB format, served as the protein receptor. At the same time, download the 2D structure of Quercetin from the PubChem database, choose to save it as “SDF” format, and use OpenBabel (version 2.4.1) software to convert it to PDB format as a small molecule ligand. Using PyMOL software to remove water molecules and original ligands from protein molecules. Then, AutoDockTools (version 1.5.6) software was used to convert the PDB format files of proteins and small molecules into PDBQT format, including some operations: hydrogenation, charge calculation, atom type addition, determination of torque center (root), etc. Finally, the protein receptor and the small molecule ligand are docked by AutoDock vina (version 1.1.2). The size of the docking binding energy of the two indicates the strength of the binding activity. Small molecule ligands can bind spontaneously, and the smaller the binding energy, the better the binding activity and the stronger the binding stability. Pymol (version 2.2.0) software was used to visualize the docking results with minimal receptor-ligand binding energy.

### Molecular docking verification-discovery studio

In order to further improve the reliability of predicted core targets, we used Discovery Studio 2019 Client software (protein structure analysis software, widely used in molecular docking) to remove water molecules, hydrogenation, apply forcefield, clean protein and other operations on the core targets, and then obtain macromolecular receptors. Hydrogenation and apply forcefield are performed on the active ligand of the core target. The macromolecular receptor is molecularly docked with the treated active ligand, and the RMSD value is obtained (RMSD represents the structural difference between two molecules (or between two states of the same molecule), a smaller value indicates a more accurate docking method). After the reliability verification of the docking software, we began to do molecular docking between the core target and the drug. Before molecular docking, we performed operations such as removing water molecules, deleting ligand groups, hydrogenation, and cleaning proteins for the core targets, and preparing ligands for small molecule drugs.

### Single-cell RNA sequencing for verification

Single cell sequencing data GSE193564 was downloaded from GEO database (https://www.ncbi.nlm.nih.gov/geo/query/acc.cgi?acc=GSE193564), which has been previously uploaded by our team. Then we analyzed the expression changes of these core targets at different time points after irradiation in rats.

### Statistical analysis

The bar chart, string chart and violin chart in this paper were analyzed and drawn in Sangerbox (http://sangerbox.com/home.html). Sangerbox is an online analysis tool developed based on R language, which has been widely recognized by peers (22).

## Results

### Dasatinib and radiation skin ulcer target identification

**Figure 1:**
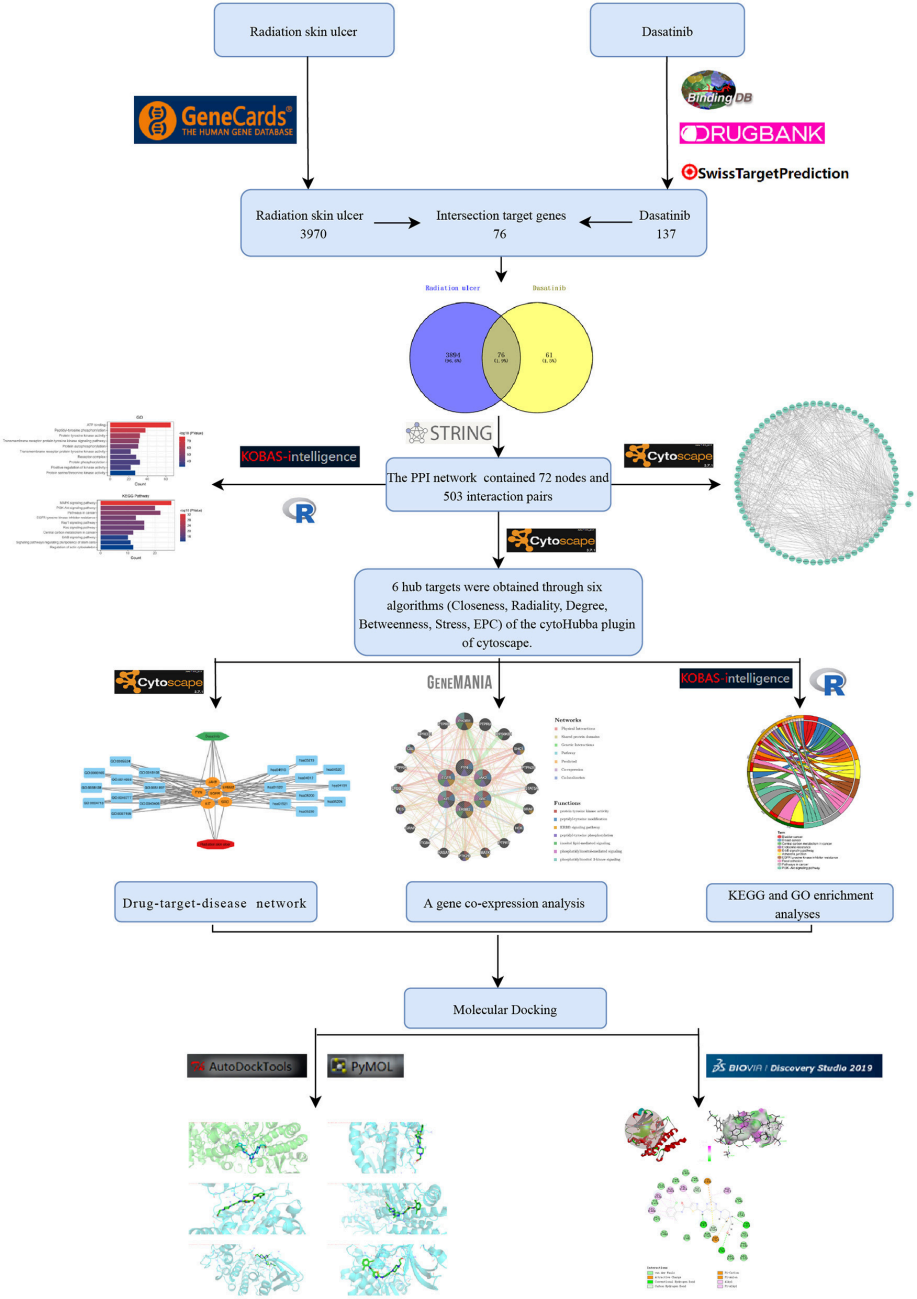
The design flow chart of this study.

**Figure 2:**
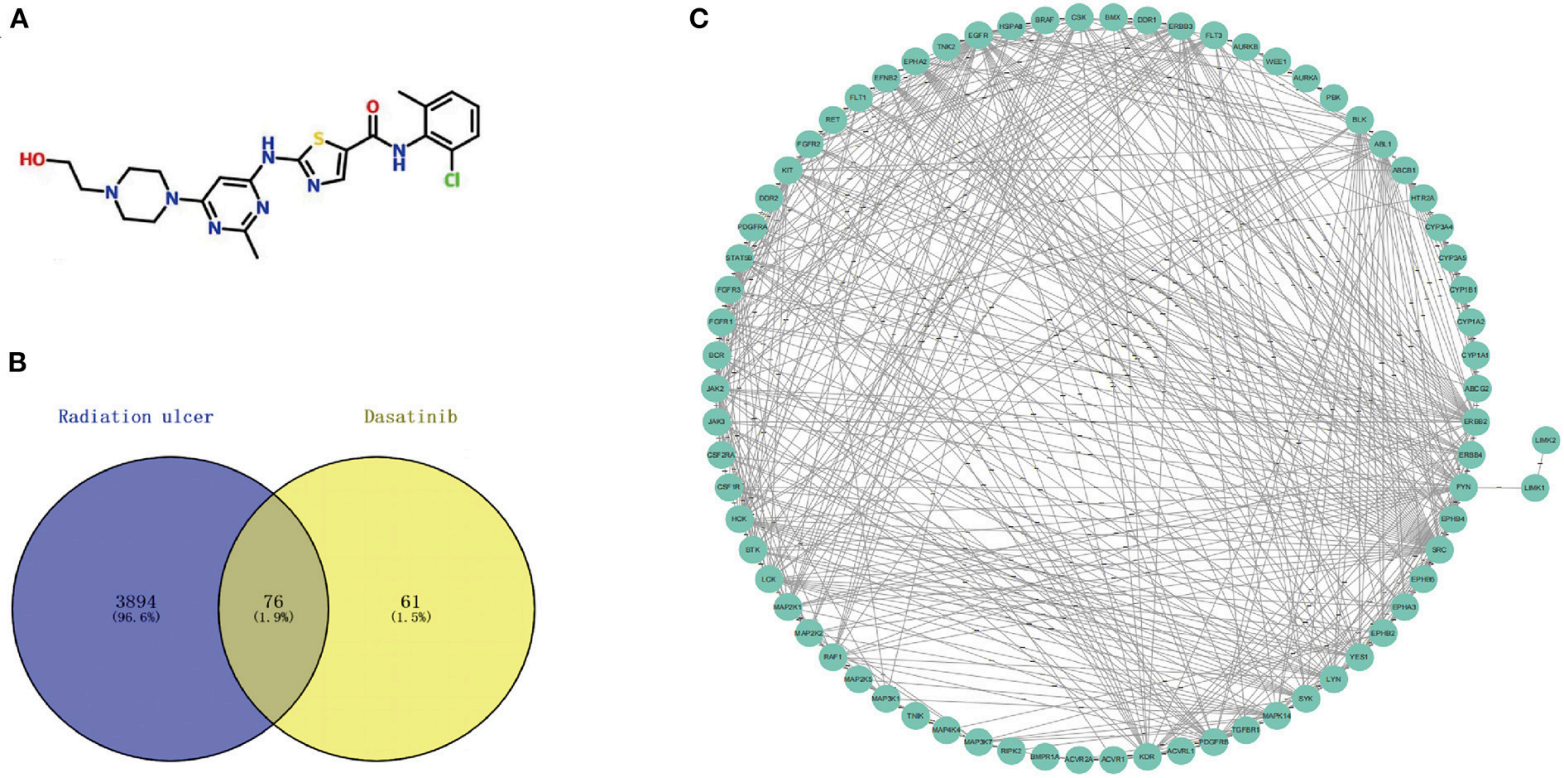
**(A)** Chemical structures of dasatinib. **(B)** A total of 76 cross targets were identified by Venn diagram. **(C)** PPI network of cross targets was constructed by using STRING database and Cytoscape software.

The flow chart of this study design is shown in Figure 1. The chemical structures of Dasatinib are shown in Figure 2A. A total of 3,970 radiation skin ulcer-related pathological targets were obtained from the GeneCards database (Supplementary Table 1). A total of 216 Dasatinib targets were obtained from STP, Drugbank and Binding DB databases (Supplementary Tables 2–4), and a total of 137 targets were obtained after screening and deduplication.

### Analysis of functional characteristics of intersection targets

**Figure 3:**
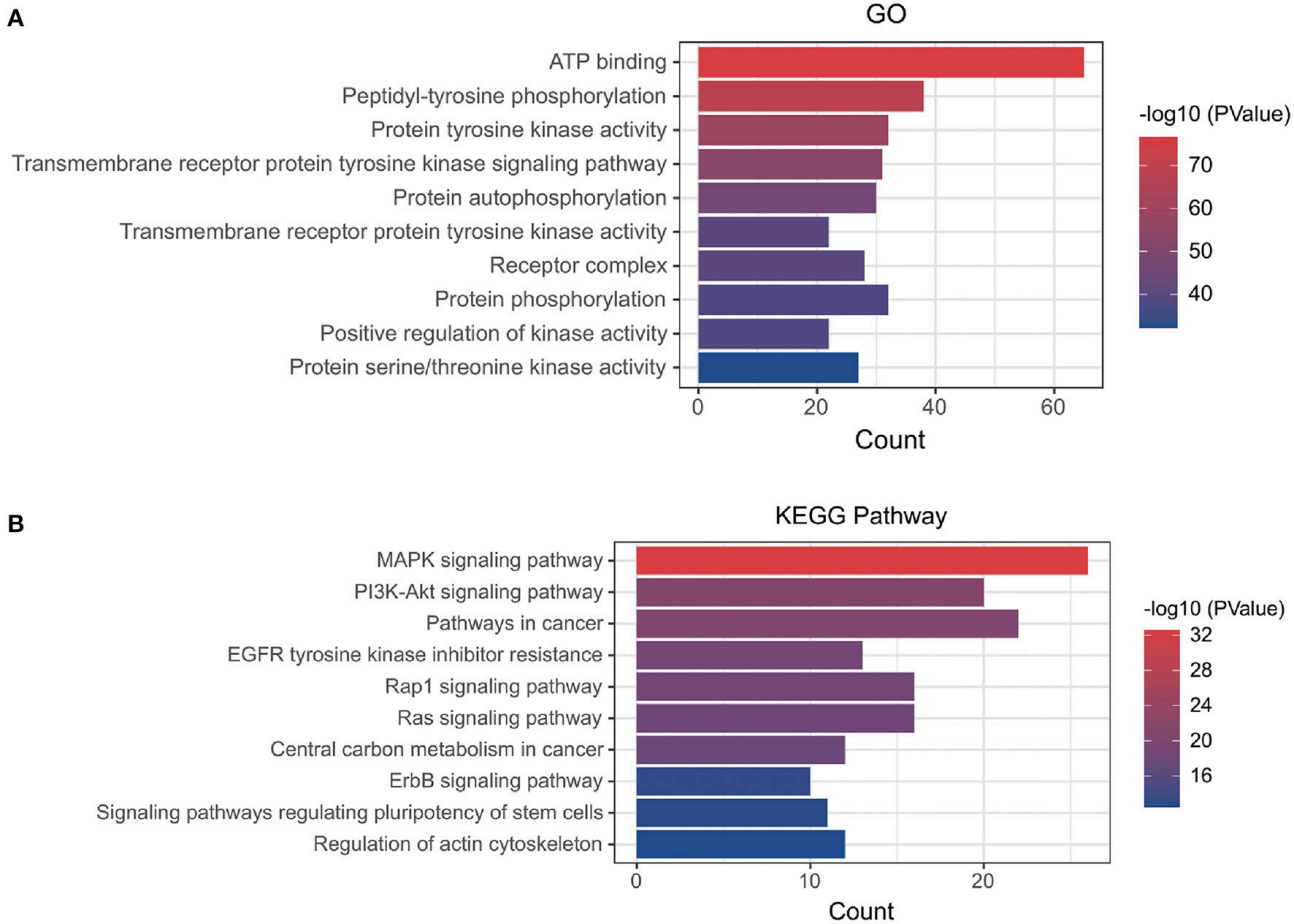
**(A)** GO and **(B)** KEGG enrichment analysis of potential targets, Histogram show the enrichment data of the first 10 terms.

Using the online tool Draw venn diagram to draw a Venn diagram for the targets related to Dasatinib activity against radiation skin ulcer, a total of 76 intersection targets were obtained (Figure 2B; Supplementary Table 5). A PPI network with a protein interaction score > 0.4 was generated from the STRING database (Figure 2C), which contains 72 nodes and 503 interaction pairs. To determine the biological functions of the intersection targets, we performed GO and KEGG pathway enrichment analysis. Figure 3 presents the top 10 meaningful enrichment results, respectively. GO analysis results show that these genes were mainly enriched in ATP binding, peptidyl-tyrosine phosphorylation, protein tyrosine kinase activity, transmembrane receptor protein tyrosine kinase signaling pathway, protein autophosphorylation, receptor complex, transmembrane receptor protein tyrosine kinase activity, protein phosphorylation, positive regulation of kinase activity and protein serine/threonine kinase activity. In terms of KEGG Pathway, these genes were mainly enriched in MAPK signaling pathway, PI3K-Akt signaling pathway, Pathways in cancer, EGFR tyrosine kinase inhibitor resistance, Rap1 signaling pathway, Ras signaling pathway, Central carbon metabolism in cancer, ErbB signaling pathway, Signaling pathways regulating pluripotency of stem cells and Regulation of actin cytoskeleton.

### Identification and analysis of core targets

**Figure 4:**
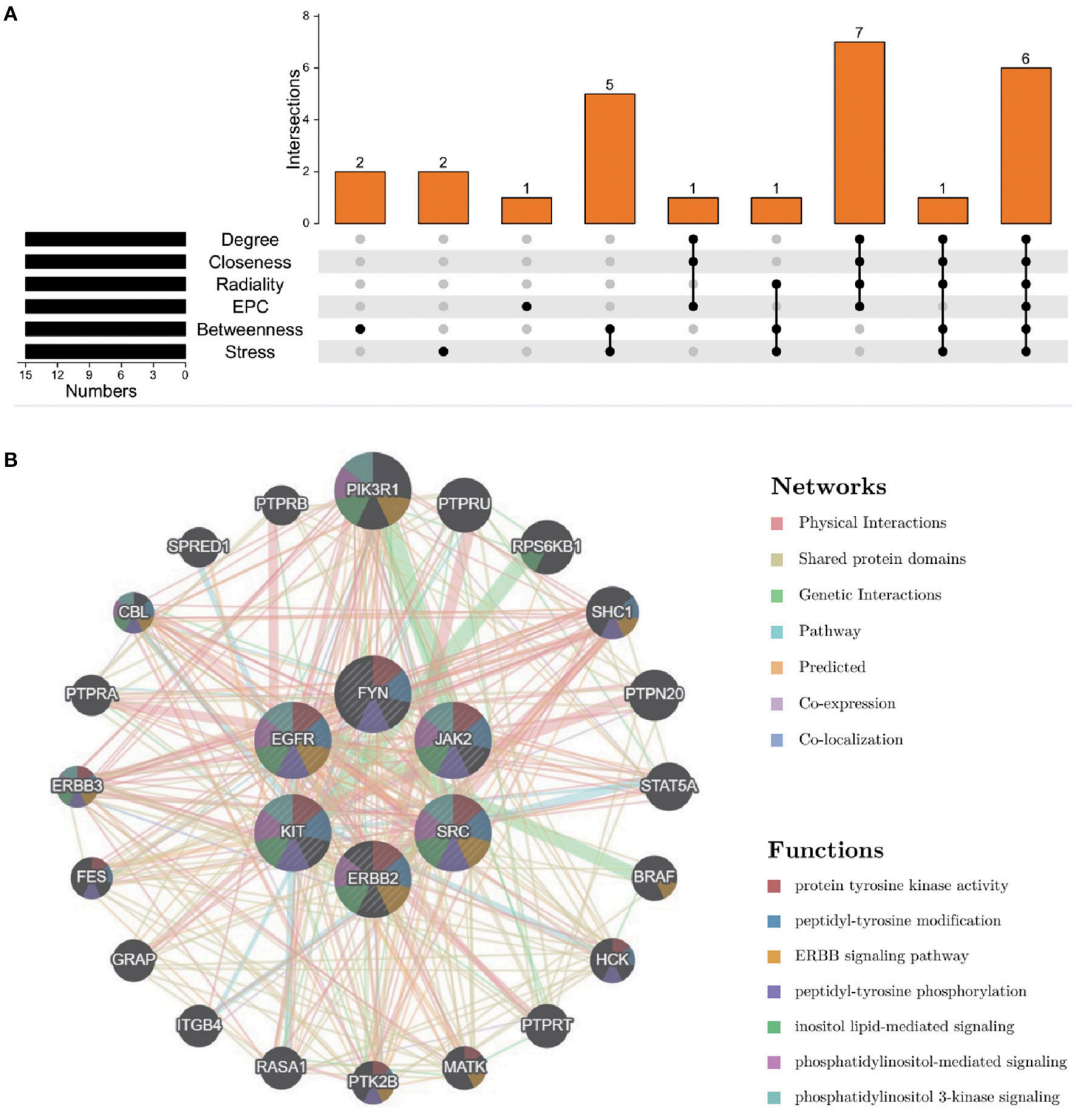
**(A)** The Venn map drawn show that 8 core targets were screened by 6 algorithms. **(B)** Core targets and its coexpression genes were analyzed by GeneMANIA.

**Figure 5:**
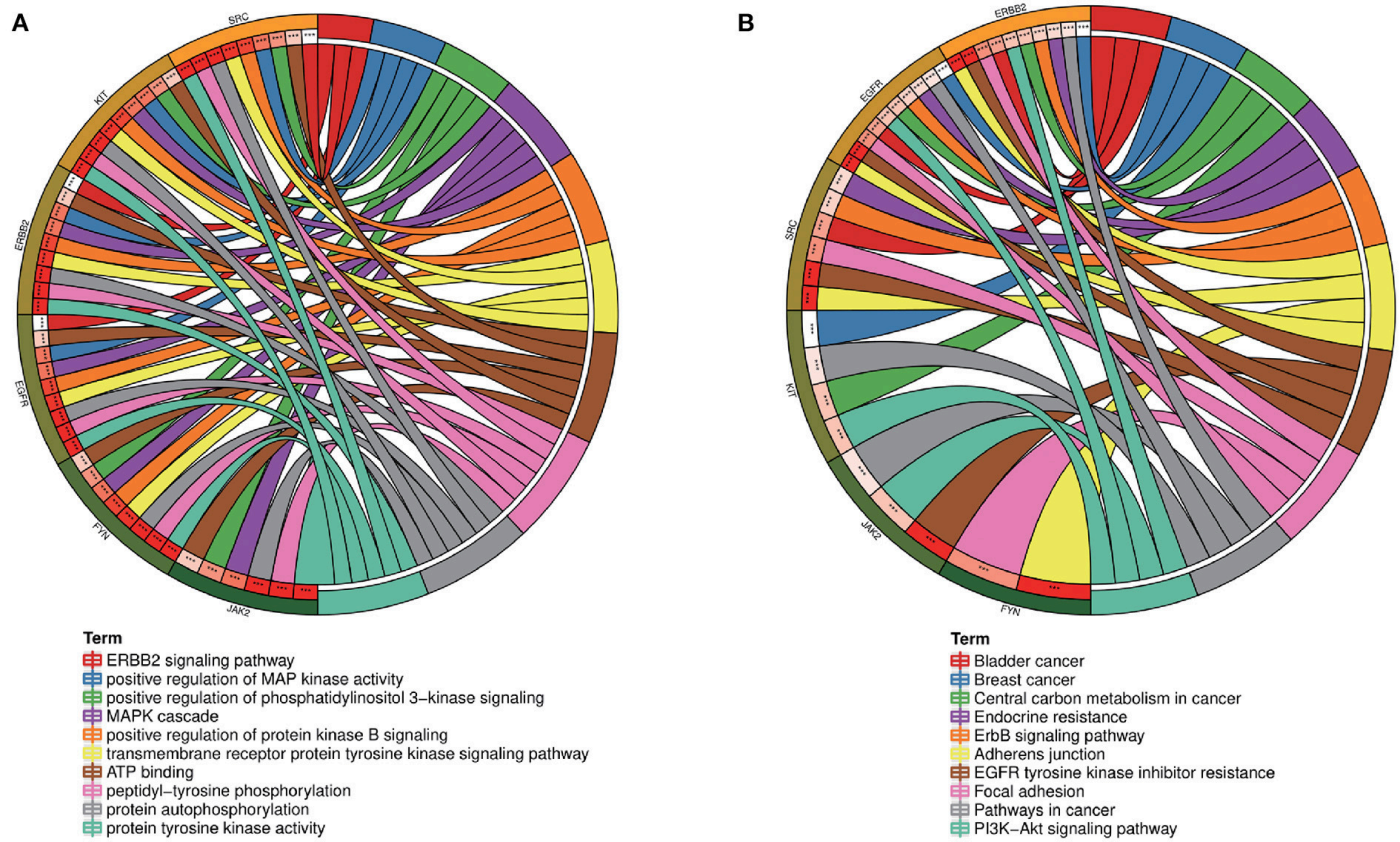
**(A)** GO and **(B)** KEGG enrichment analysis of core targets showed the top 10 enrichment data. The outermost circle is term on the right and the inner circle on the left represents the significant P-value of the corresponding pathway of the gene.

**Figure 6:**
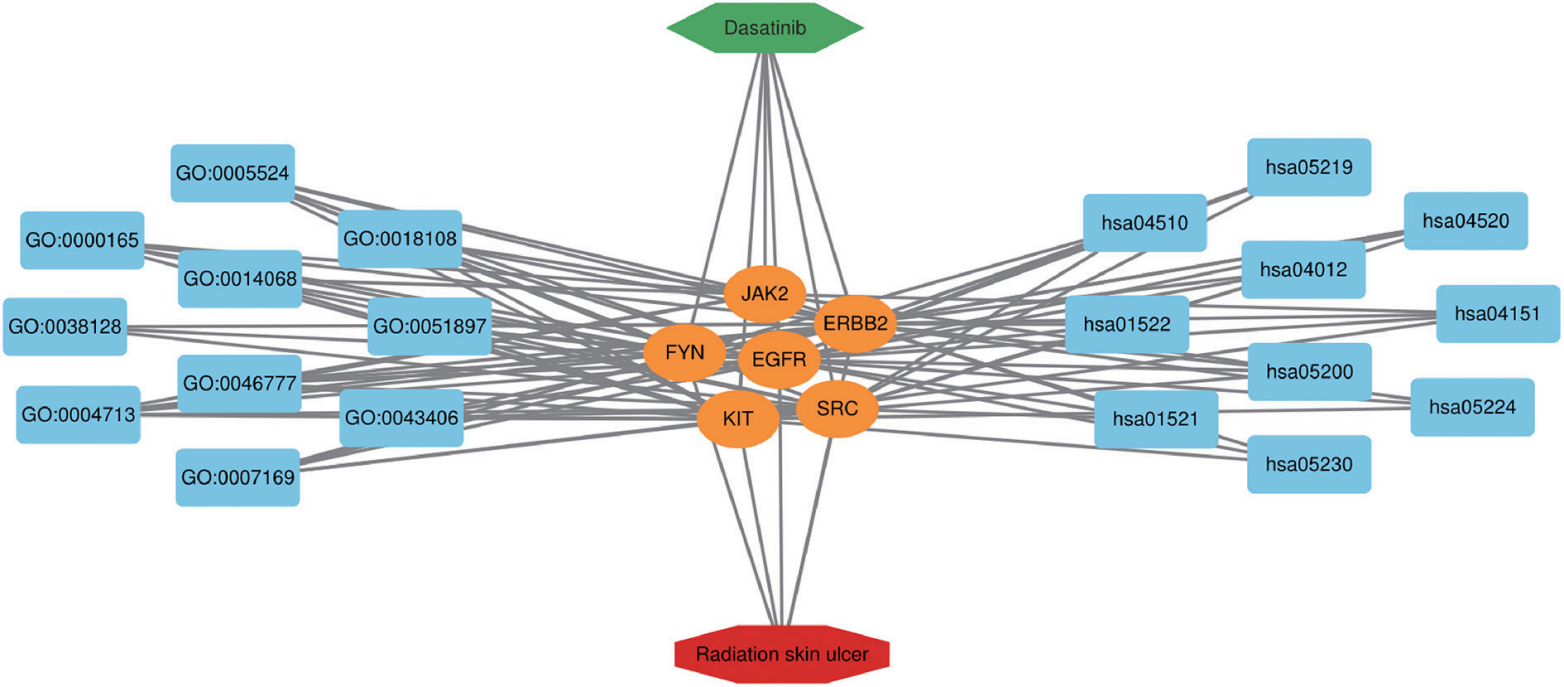
Dasatinib-target-radiation ulcers detailed interaction network diagram. The blue rectangle on the left represents the first 10 BP, and the blue rectangle on the right represents the first 10 KEGG pathways. The orange oval in the middle represents the core targets.

To further explore the core targets of Dasatinib activity against radiation skin ulcer, we calculated the top 15 core targets using 6 algorithms in cytohubba (Table 1). By taking the intersection, a total of 6 core targets were obtained, including EGFR, ERBB2, FYN, JAK2, KIT, and SRC (Figure 4A). Table 2 shows their full names and functions. Co-expression network and function analysis of core targets based on GeneMANIA database showed a complex PPI network with the physical interactions of 38.72%, shared protein domains of 26.13%, genetic interactions of 14.21%, pathway of 9.27%, predicted of 8.87%, co-expression of 2.30%, and co-localization of 0.49% (Figure 4B). To further explore the biological functions of core targets, we analyzed and displayed the top 10 GO and KEGG pathway enrichment analysis results (Figure 5). Similar to the previous results, GO analysis results show that these genes were mainly enriched in protein tyrosine kinase activity, peptidyl-tyrosine phosphorylation, protein autophosphorylation, transmembrane receptor protein tyrosine kinase signaling pathway, positive regulation of protein kinase B signaling, MAPK cascade, positive regulation of MAP kinase activity, positive regulation of phosphatidylinositol 3-kinase signaling, ATP binding and ERBB2 signaling pathway. In terms of KEGG Pathway, these genes were mainly enriched in Adherens junction, EGFR tyrosine kinase inhibitor resistance, Focal adhesion, Bladder cancer, PI3K-Akt signaling pathway, Central carbon metabolism in cancer, ErbB signaling pathway, Endocrine resistance, Pathways in cancer and Breast cancer. Finally, a network visualization of dasatinib activity against radiation skin ulcer targets and an interaction diagram for core target-related pathways were generated by Cytoscape software (Figure 6).

**Table 1:** The top 15 core targets rank in cytoHubba.

**Degree**	**Closeness**	**Radiality**	**EPC**	**Betweenness**	**Stress**
SRC	SRC	SRC	SRC	EGFR	EGFR
EGFR	EGFR	EGFR	EGFR	SRC	SRC
FYN	FYN	FYN	FYN	FYN	ABCB1
ERBB2	ERBB2	ERBB2	ERBB2	ABCB1	FYN
JAK2	JAK2	JAK2	JAK2	MAPK14	MAPK14
YES1	KIT	ABL1	YES1	KDR	ERBB2
ERBB3	ABL1	KDR	ABL1	ERBB2	ABCG2
STAT5B	ERBB3	ERBB3	STAT5B	ABCG2	KDR
KIT	YES1	KIT	KIT	EPHB2	MAP3K1
ABL1	STAT5B	YES1	LCK	MAP3K1	JAK2
HCK	HCK	HCK	HCK	MAP3K7	RAF1
LCK	LCK	MAPK14	ERBB3	JAK2	MAP2K1
LYN	KDR	LCK	LYN	LIMK1	KIT
PDGFRB	LYN	LYN	PDGFRB	TGFBR1	TGFBR1
KDR	PDGFRB	STAT5B	ERBB4	KIT	EPHB2

**Table 2:** The details of the core targets.

**Gene symbol**	**Full name**	**Function**
EGFR	Epidermal growth factor receptor	EGFR is a cell surface protein that binds to epidermal growth factor. Binding of the protein to a ligand induces receptor dimerization and tyrosine autophosphorylation and leads to cell proliferation.
JAK2	Janus kinase 2	This gene product is a protein tyrosine kinase involved in a specific subset of cytokine receptor signaling pathways. It has been found to be constitutively associated with the prolactin receptor and is required for responses to gamma interferon.
FYN	FYN proto-oncogene, SRC family tyrosine kinase	This gene is a member of the protein-tyrosine kinase oncogene family. It encodes a membrane-associated tyrosine kinase that has been implicated in the control of cell growth.
ERBB2	Erb-B2 receptor tyrosine kinase 2	This gene encodes a member of the epidermal growth factor (EGF) receptor family of receptor tyrosine kinases. It does bind tightly to other ligand-bound EGF receptor family members to form a heterodimer, stabilizing ligand binding and enhancing kinase-mediated activation of downstream signaling pathways, such as those involving mitogen-activated protein kinase and phosphatidylinositol-3 kinase.
KIT	KIT proto-oncogene, receptor tyrosine kinase	This protein is a type 3 transmembrane receptor for MGF (mast cell growth factor, also known as stem cell factor). Mutations in this gene are associated with gastrointestinal stromal tumors, mast cell disease, acute myelogenous leukemia, and piebaldism.
SRC	Src proto-oncogene, non-receptor tyrosine kinase	This proto-oncogene may play a role in the regulation of embryonic development and cell growth. The protein encoded by this gene is a tyrosine-protein kinase whose activity can be inhibited by phosphorylation by c-SRC kinase.

### Molecular docking-AutoDock vina

**Figure 7:**
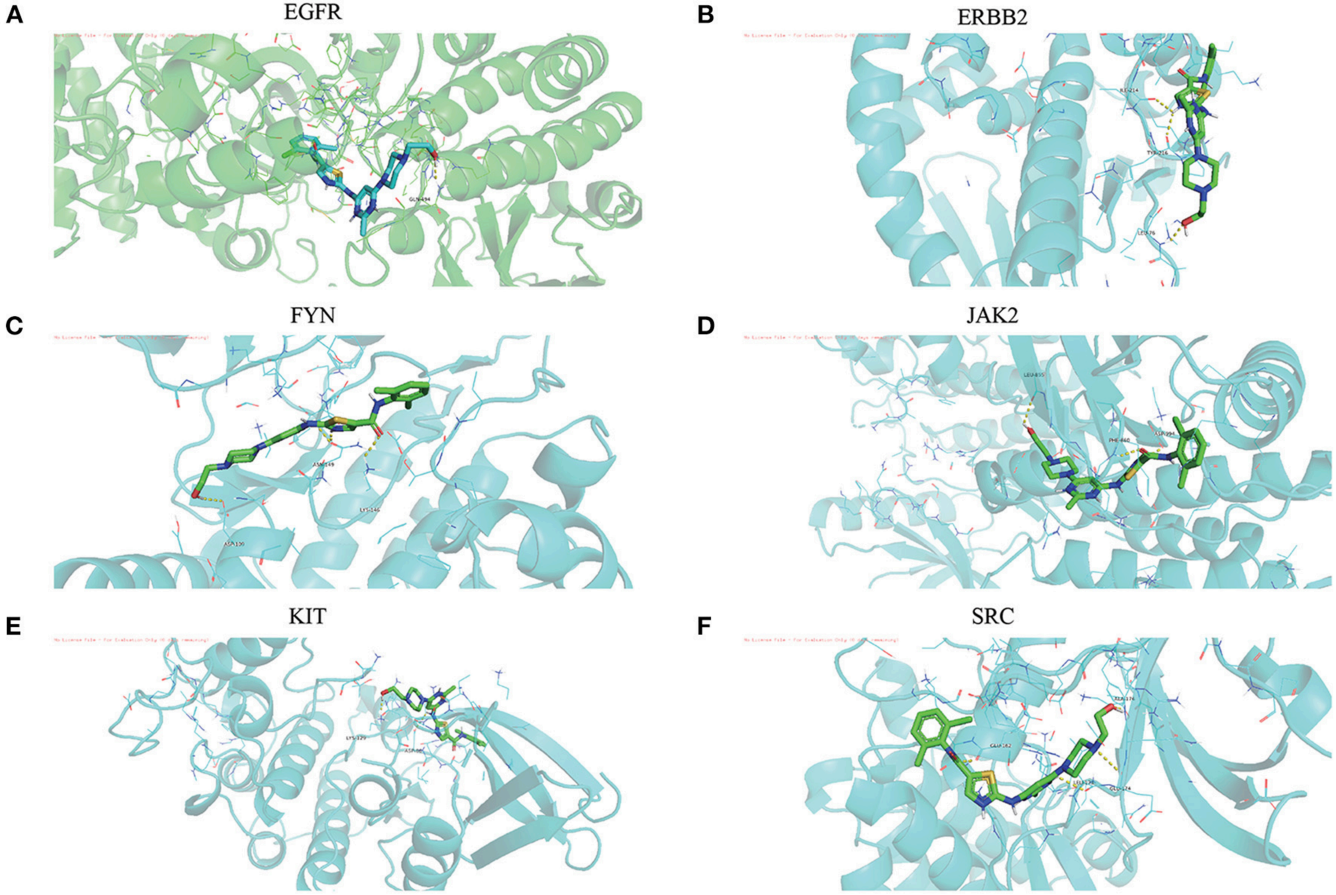
**(A-F)** The visualization of dasatinib docking with six core targets. The docking results of the core targets are displayed by PyMOL software, and the yellow dotted line represents the interaction line between the target and the compound.

The Dasatinib was molecularly docked with the core genes EGFR, ERBB2, FYN, JAK2, KIT, and SRC, respectively. According to the molecular docking binding energy, each histone-ligand could spontaneously bind (binding energy <0 kcal/mol). The binding energies of EGFR, ERBB2, FYN, JAK2, KIT, and SRC were all ≤ −5 kcal/mol, indicating good protein-ligand binding. See Figure 7 for the visualization of molecular docking. The molecular docking details are shown in Table 3.

**Table 3:** Molecular docking of dasatinib.

**Hub**	**Compound**	**PDB ID**	**Binding**	**Hydrogen**
**target**			**energy**	**bonding**
			**(KJ/mol-1)**	**residues**
EGFR	Dasatinib	5J87	−8.2	GLN-494
ERBB2	Dasatinib	3R91	−7.0	ILE-214, TYR-216, LEU-76
FYN	Dasatinib	4QMS	−7.9	ASN-149, LYS-146, ASP-109
JAK2	Dasatinib	6X8E	−9.2	LEU-855, PHE-860, ASP-994
KIT	Dasatinib	6Q4G	−9.5	LYS-129, ASP-86
SRC	Dasatinib	5VCY	−8.0	ALA-176, GLU-162, LEU-173, HLU-174

### Molecular docking-discovery studio

**Figure 8:**
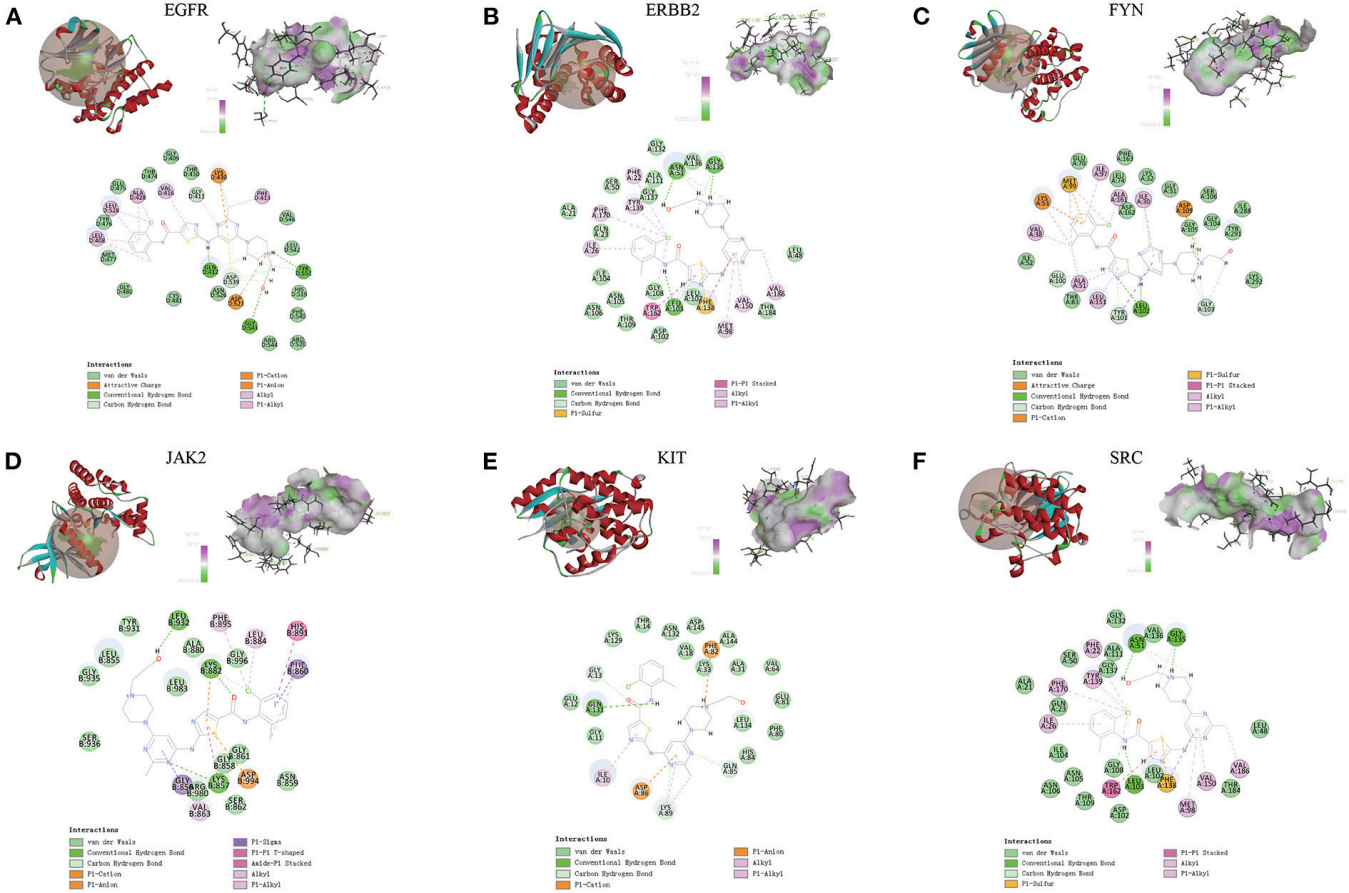
**(A-F)** Molecular docking results of EGFR, ERBB2, FYN, JAK2, KIT and SRC with dasatinib. (Upper left) Dasatinib docking model with the core targets. (Top right) The interaction diagram of dasatinib and the core targets hydrogen bond residues, the greener the color, the stronger the interaction force, and the more purple the color, the weaker the interaction force. (Bottom) Various interactions of dasatinib with core targets, each color in the circle corresponds to a mode of action.

Compared with the original crystal structures, the RSMD values of EGFR, ERBB2, FYN, JAK2, KIT, and SRC were 1.4574, 1.1103, 1.4139, 0.5710, 0.5868, 1.1455A. The ligand conformation in the original crystal structure of the core protein basically overlaps with the docked ligand conformation, and RMSD <2A indicates that the calculation method can accurately predict the binding mode of the original ligand. The molecular docking results of EGFR, ERBB2, FYN, JAK2, KIT, and SRC with Dasatinib are shown in Figure 8. AutoDock vina and Discovery Studio use different algorithms and scoring functions, which may cause differences in screening results. Combining two different algorithms, we believe that EGFR, ERBB2, FYN, JAK2, KIT, and SRC are likely to become core targets.

### Single-cell RNA sequencing

**Figure 9:**
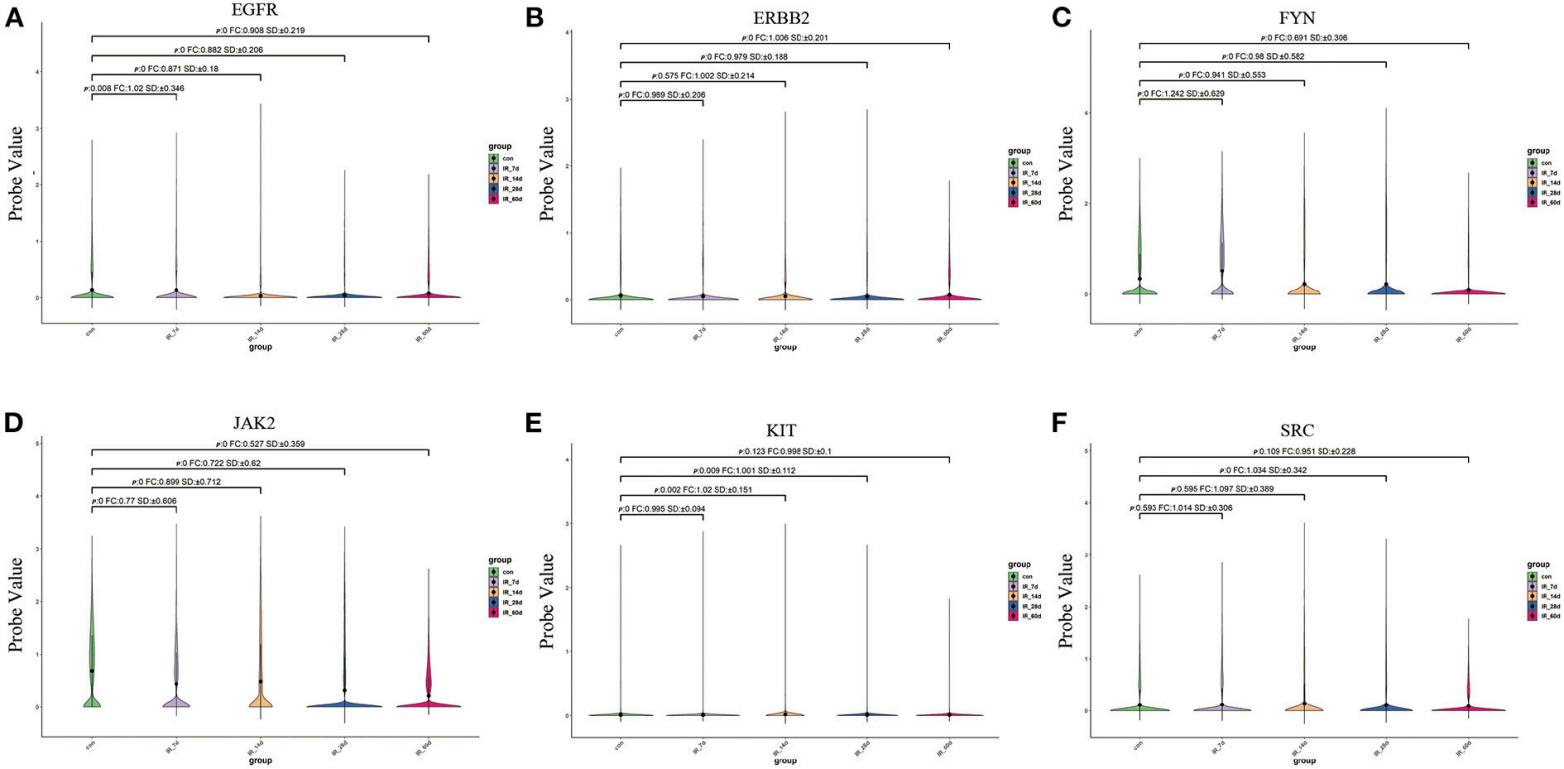
**(A–F)** Expression of six core targets in rat skin tissue after irradiation was examined by single-cell RNA sequencing. FC > 1 represents up-regulation, and FC <1 represents down-regulation.

We extracted the expression values of the six core targets in control skin and post-irradiated skin. *T*-test was used to verify whether there was a significant difference between the groups. The results showed that the six core targets had significant expression changes at different time points after irradiation (Figure 9; Supplementary Table 6).

## Discussion

Radiation-induced skin damage can be roughly divided into four stages: immediate (0–2 weeks), early (2–8 weeks), mid-term (6–52 weeks), and late (over 40 weeks) according to the time after exposure. In addition, radiation damage response is dose-dependent, and the National Cancer Institute has identified four skin toxicity grades for radiation dermatitis. The most common immediate reaction (dose as low as 2 Gy) is transient erythema (grade 1), which is an inflammatory response with changes in cellular permeability and histamine release (23–25). Early reactions (at doses of 3 to 6 Gy) included temporary alopecia, erythematous reactions, and moderate tissue edema (grade 2). Doses greater than 10 Gy can cause more severe consequences, such as dry or moist peeling (grade 3) (24). Intermediate responses (dose greater than 15 Gy) can include skin atrophy, necrosis, and ulceration (grade 4). Late reactions are characterized by telangiectasia, further thinning of the skin, and deepening of the ulcer (23).

Ionizing radiation causes skin cell death through a variety of pathways, including induction of apoptosis, mitotic disorders, and cellular senescence (26). Reactive oxygen species (ROS) induced by ionizing radiation can induce damage to DNA, endoplasmic reticulum, and mitochondria (27, 28). Recent studies have shown that exposure to radiation also leads to impairment and damage to the immune system, triggering the production of inflammatory cytokines and leading to a systemic inflammatory state (29–31). As previously described, impaired vascular integrity, resulting in transient intra-tissue hemorrhage, promoted cutaneous wound healing in mice with combined platelet GPVI and CLEC2 deficiency (32). Thus, a short-term loss of vascular integrity following dasatinib administration, especially at 5 mg/kg, allowing plasma-derived molecules (including fibrinogen, growth factors, and cytokines) to enter the wound to promote tissue repair (19, 33, 34). High concentrations of dasatinib inhibited platelet aggregation and tyrosine phosphorylation *in vitro* upon thrombin stimulation (19). More importantly, inhibition of thrombin may affect the repair process, as synthetic thrombin peptides have been shown to promote angiogenesis and promote wound healing in animal skin (35, 36). In addition, high-dose or prolonged exposure to dasatinib may cause vessel wall rupture through its direct effects on endothelial cells (20, 37, 38).

This study focused on the potential mechanism of action of dasatinib in the treatment of radiation ulcers. Network analysis increases the understanding of multiple mechanisms of drug action, while systems pharmacology may provide new targets for drug discovery. By intersecting the potential targets of dasatinib and radiation ulcers in the bioinformatics database, 137 potential therapeutic targets of dasatinib for radiation ulcers were finally obtained. Subsequently, we constructed a PPI network with potential therapeutic targets and further screened six core targets (EGFR, ERBB2, FYN, JAK2, KIT, and SRC).

EGFR is widely expressed in normal skin tissues, such as epidermis, sebaceous glands, glands, eccrine glands, and dendritic cells (39), and plays an important role in the development and physiology of normal epidermis (40). EGFR may be activated through signaling pathways such as MAPK, STATs and PI3K (41). Inhibition of EGFR increases the expression of the cyclin-dependent kinase inhibitor p27-KIP1 (42–44), which leads to cell cycle arrest in the G1 phase of keratinocytes (45). STAT3 is activated by EGFR signaling and is a key molecule in maintaining skin homeostasis. Loss of STAT3 expression in transgenic mice affects wound healing (46), showing impaired hair follicle development and hair growth and a strong inflammatory response (47–49). Children with loss of EGFR expression develop lifelong inflammation in the skin, gut, and lungs, leading to early infant mortality, highlighting the importance of EGFR signaling in establishing and maintaining tissue homeostasis (50). Furthermore, the EGFR signaling pathway is critical for the maintenance of bone precursor cells and the formation of new bone (51). In mice, EGFR-specific inhibitors or inhibition of EGFR in osteoprogenitors and osteoblasts results in a reduction in the number of bone mesenchymal progenitors leading to bone loss (52). It has recently been reported that EGFR signaling inhibits cellular and tissue senescence (53–55). The EGFR signaling pathway in osteoprogenitor cells plays an important role in preventing cellular senescence and regulating cortical bone metabolism. In addition, ERBB2, a member of the EGFR family, is expressed in the epidermis and hair follicle root sheath (56), and overexpression of ERBB2 has also been found in skin cancer patients (57). Furthermore, in a mouse model overexpressing ERBB2, the epidermis and follicles of the skin are hyperproliferative (58–61). All of the above studies suggest that dasatinib may be effective in the treatment of radiation ulcers by targeting EGFR signaling.

Few studies have been performed on other core targets related to radiation ulcers and deserve further consideration in future studies. Given that radiation ulcers are still a major problem facing the global medical system, our study is the first to use network pharmacology to explore the potential core targets and mechanisms of action of dasatinib against radiation ulcers. Their feasibility and possible docking sites are verified by molecular docking technology, which will provide a reference for the study of the molecular mechanism of dasatinib in the treatment of radiation ulcers. However, our study also has certain limitations. First, the core targets and pharmacological mechanisms of dasatinib in the treatment of radiation ulcers need to be verified *in vivo* and *in vitro* models, which will be the focus of our future research. Secondly, whether the pharmacological effects of dasatinib are as helpful to human patients as in animal and cell experimental models is also a problem that we need to solve. In follow-up studies, we will further verify the feasibility of treating radiation ulcers with core targets in animal and clinical trials.

## Conclusion

Overall, this is the first time that bioinformatics methods such as network pharmacology and molecular docking have been applied to systematically explore the pharmacological and molecular mechanisms of dasatinib in the treatment of radiation skin ulcers. Dasatinib may promote the healing of radiation skin ulcers through EGFR tyrosine kinase inhibitor resistance, PI3K-Akt signaling pathway and ErbB signaling pathway. During treatment, EGFR, ERBB2, FYN, JAK2, KIT, and SRC were its main potential pharmacological targets, and the molecular docking results confirmed our conclusion. The identified potential targets and pathways may be validated in preclinical studies before being used in clinical therapy.

## Data availability statement

The datasets presented in this study can be found in online repositories. The names of the repository/repositories and accession number(s) can be found in the article/Supplementary material.

## Ethics statement

The animal study was reviewed and approved by Ethics Committee of the Second Affiliated Hospital of Chengdu Medical College.

## Author contributions

WS and DY: conceptualization. XiC, XuC, and WZ: data curation. WS, DL, and XiC: formal analysis and methodology. DY, DL, and XuC: supervision. WS, XuC, and DL: writing (review and editing). The final manuscript read and approved by all authors. All authors contributed to the article and approved the submitted version.

## Funding

This work was supported by the National Natural Science Foundation of China (32071238), the Fundamental Research Funds for the Central Universities and Young Talent Program of China National Nuclear Corporation (CNNC2021136), the Natural Science Project of Chengdu Medical College (CYZYB21-07), and the Medical Research Project of Chengdu 2021 (2021085).

## Conflict of interest

The authors declare that the research was conducted in the absence of any commercial or financial relationships that could be construed as a potential conflict of interest.

## Publisher's note

All claims expressed in this article are solely those of the authors and do not necessarily represent those of their affiliated organizations, or those of the publisher, the editors and the reviewers. Any product that may be evaluated in this article, or claim that may be made by its manufacturer, is not guaranteed or endorsed by the publisher.

## Supplementary material

Supplementary Table 1 The pathological targets of radiation skin ulcer form GeneCards database.

Supplementary Table 2 The dasatinib pharmacological targets form SwissTargetPrediction database.

Supplementary Table 3 The dasatinib pharmacological targets form Drugbank database.

Supplementary Table 4 The dasatinib pharmacological targets form Binding DB database.

Supplementary Table 5 The targets related to dasatinib activity against radiation skin ulcer.

Supplementary Table 6 Expression values of core targets in cells of rats at different time points after irradiation.

## Abbreviations

DEGs: differentially expressed genes
WGCNA: weighted gene co-expression network analysis
PPI: protein-protein interaction
Treg: regulatory T cells
GEO: Gene Expression Omnibus
TBSA: total burn surface area
TOM: topological overlap matrix
GS: gene significance
GO: Gene ontology
KEGG: Kyoto Encyclopedia of Genes and Genomes
MCODE: molecular complex detection technology
ImmuCellAI: The Immune Cell Abundance Identifier
HLA-G: human leukocyte antigen G.
